# Biomaterials for Cleft Lip and Palate Regeneration

**DOI:** 10.3390/ijms20092176

**Published:** 2019-05-02

**Authors:** Marcela Martín-del-Campo, Raúl Rosales-Ibañez, Luis Rojo

**Affiliations:** 1 Facultad de Estomatología, Universidad Autónoma de San Luis Potosí, Av. Dr. Salvador Nava No. 2, Zona Universitaria, San Luis Potosí (S.L.P.) 78290, Mexico; mar_tin53@hotmail.com; 2 Consejo Superior de Investigaciones Científicas, Instituto de Ciencia y Tecnología de Polímeros, Calle Juan de la Cierva, 3, 28006 Madrid, Spain; 3 Laboratorio de Ingeniería Tisular y Medicina Traslacional, Facultad de Estudios Superiores Iztacala, Universidad Nacional Autónoma de Mexico, Avenida de los Barrios N 1, Iztacala Tlalnepantla, Estado de Mexico 54090, Mexico; rosales_ibanez@unam.mx; 4 Consorcio Centro de Investigación Biomédica en Red CIBER-BBN, Calle Monforte de Lemos S/N, 28029 Madrid, Spain

**Keywords:** cleft palate, cleft lip, regenerative medicine, bone, craniofacial defects, orofacial disorders, musculoskeletal tissue engineering

## Abstract

Craniofacial bone defect anomalies affect both soft and hard tissues and can be caused by trauma, bone recessions from tumors and cysts, or even from congenital disorders. On this note, cleft/lip palate is the most prevalent congenital craniofacial defect caused by disturbed embryonic development of soft and hard tissues around the oral cavity and face area, resulting in most cases, of severe limitations with chewing, swallowing, and talking as well as problems of insufficient space for teeth, proper breathing, and self-esteem problems as a consequence of facial appearance. Spectacular advances in regenerative medicine have arrived, giving new hope to patients that can benefit from new tissue engineering therapies based on the supportive action of 3D biomaterials together with the synergic action of osteo-inductive molecules and recruited stem cells that can be driven to the process of bone regeneration. However, few studies have focused on the application of tissue engineering to the regeneration of the cleft/lip and only a few have reported significant advances to offer real clinical solutions. This review provides an updated and deep analysis of the studies that have reported on the use of advanced biomaterials and cell therapies for the regeneration of cleft lip and palate regeneration.

## 1. Introduction

**Figure 1:**
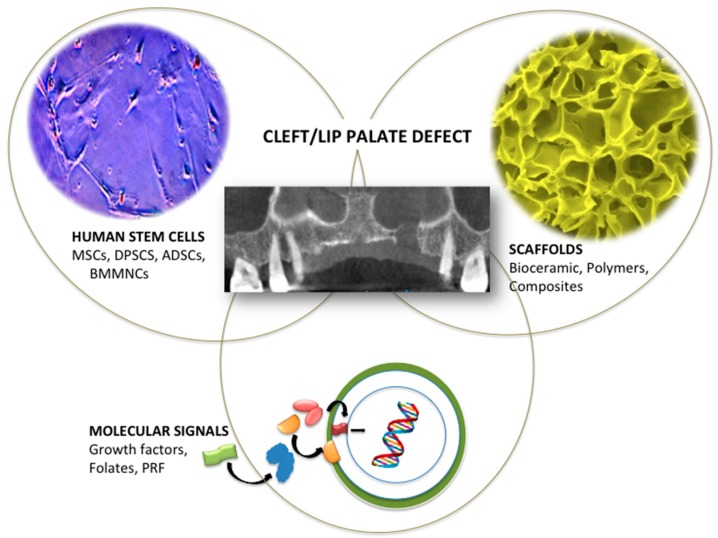
Human stem cells, biomimetic scaffolds, and regenerative molecule signals as fundamental pieces of the tissue engineering puzzle for cleft/lip palate regeneration.

Craniofacial defects generally cause significant negative impacts on the quality of life and self-esteem of those individuals with musculoskeletal dysfunctionalities. Cleft lip, with or without cleft palate (CL/P), is the most prevalent congenital craniofacial defect caused by disturbed embryonic development of soft and hard tissues around the oral cavity and face area [1]. Current treatments for this orofacial condition generally demand early surgery and face reconstruction procedures that may be revised during childhood and infancy, causing a great number of patient complaints and economic burden to health systems that need to be minimized. Due to these reasons, alveolar cleft reconstruction has been considered one of the most controversial surgical procedures and less invasive therapies have being demanded since the beginning of the 20th century [2]. Fortunately, tissue engineering is rapidly providing successful regenerative therapies to several musculoskeletal conditions based on the synergic triad of using functional biomaterials, in conjunction with the vehiculization and local delivery of bioactive regenerative molecules and guided or recruited stem cells (Figure 1) that can modulate the etiopathogenesis of the disease and its prevalence by promoting the missing self-repairment mechanisms of affected tissues, thus improving the life conditions of affected patients. The functional reconstruction of highly vascularized bones, such as the craniofacial area, is a key challenge in bone tissue engineering, since it depends fundamentally on a well-organized hierarchical vascular network. The cell survival and viability, as well as the elimination of metabolic waste are in charge of the supply of oxygen and nutrients carried out by the blood vessels, in this way, the restoration of the neovasculature contributes to improve bone functionality [3]. Scaffold materials should allow vascular regeneration in a fundamental way as well as provide structure, osteonduction and osteoconduction characteristics when applied in the field of craniofacial regeneration [4]. Thus, accordantly with different authors, an ideal bone construction should combine a weightbearing rigid scaffold design, a porous structure that mimics the bone architecture, and cell-laden materials that favor new vascular formation [5]. The pore size and shape of a particular biomaterial play a key role in vascular ingrowth [6]. However, the size of the interconnections seems to be more important for the vascularization of a scaffold when compared with the pore size [7]. As such, fabrication designs, biocompatibility characteristics, porosity and matrix density are of critical consideration [3]. Despite the importance of this knowledge in the study of the craniofacial defect regeneration, there have been few studies on CL/P that deepen in assays on the neovascularization of tissues through the proposal of new materials. This review provides an updated and deep analysis of the studies that have reported on the use of advanced biomaterials and cell therapies for the regeneration of cleft lip and palate regeneration.

### 1.1. Etiopathogenesis of Orofacial Cleft

Cleft palate (CL/P) malformation occurs as a result of the non-fusion of the primary palate during the fourth and 12th weeks of gestation [2,8]. During this period, the embryo undergoes rapid changes in shape and growth as the brain expands simultaneously for the formation of the branchial arches responsible for the development of the face and the cranium. Alar structures of the nose are formed by the lateral nasal process while, during the mandibular processes that take place during the eighth week, the shelves ascend above the tongue and then fuse, forming the secondary palate completing the formation of the jaw, the upper lip, alveolus, and primary palate [2]. Like any other structural formation in the human body, the entire process is guided by a precise synchronization and balance of cell adhesion, proliferation, and differentiation, regulated by cell signaling molecules from which the family of transforming growth factor beta (TGF-b), fibroblast growth factors (FGFs), bone morphogenic proteins (BMPs), and sonic hedgehog (SHH) [2,9] stands out. Dysfunctions on these pathways, mediated by gene regulation, are responsible for most of the common presentations of human maxillary alveolar cleft, a bony oronasal communication lined by epithelialized mucosa and partially erupted or unerupted teeth within the cleft [10].

Environmental factors or maternal metabolic imbalances and infections during embryogenesis ultimately contribute to the etiology of musculoskeletal dysfunctionalities being maternal folic acid deficiency during the periconceptional period or exposure to alcohol and teratogenic medications, i.e., retinoids, corticosteroids, and the anticonvulsant phenytoin and valproic acid, which is the main cause of cleft disorders [2].

### 1.2. Prevalence

**Figure 2:**
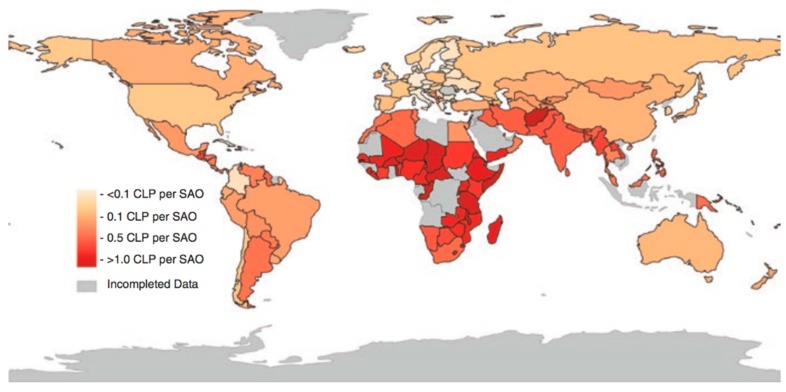
World incidence of cleft lip/palate per surgeon, anthologist, and obstetrician (SAO) in each country. Reproduced from Massenburg et al. (2018) [11] with permission from Springer ©.

Orofacial cleft conditions have been estimated to have a global annual prevalence of 7.94 cases per 10,000 live births with high variances of treated patients across regions and countries (Figure 2) [11]. In some European countries, for example, the prevalence of CL/P has been reported between 0.53 to 1.59 cases per 1000 live births [12], while the countries that have reported the highest and lowest rates were Japan (19.05) and South Africa (3.13), respectively. On the other hand, in the American continent, the overall case rate is 10.49 per 10,000 live births and this figure is surpassed by some countries in South America (i.e., Bolivia with 23.7, Ecuador with 14.96, and Paraguay with 13.3). Conversely, the lowest figures were presented in countries such as Venezuela with 7.92, Peru with 8.94, Uruguay with 9.37, and Brazil with 10.12, all for 10,000 live births [13]. Within the USA, the average prevalence of cleft lip with or without cleft palate was 7.75 per 10,000 live births, showing differences between ethnicities [14].

### 1.3. Cost at the Health, Social and Economic Level

CL/P is considered as an anatomical defect of profound aesthetic and functional impact that leads to other future alterations, and therefore may negatively impact health-related quality of life, and/or speech [12]. Individuals with clefts of the lip, palate, or alveolus often require interdisciplinary treatment into adulthood and thus they require timely and effective care. In addition, the repercussions of this disease affect the family nucleus and the social environment that in many cases may carry the financial burden of extensive treatment, and a variety of psychosocial challenges [13,15]. The economic impact of CL/P therapies on national health systems is difficult to estimate due to the number of analyses and examinations that every child born with a CL/P must go through for several years. Routine analysis of airway obstruction, in relation to feeding capacity and nutritional intake, weight and growth rates, different musculoskeletal abnormalities, genetic tests to associate syndromes and craniofacial examination to evaluate the shape of the head, ears, eyes, nose, jaws and oral cavity need to be assessed, costing up to $2.4 billion per year according to the World Health Organization [16].

## 2. Clinical Demands

**Figure 3:**
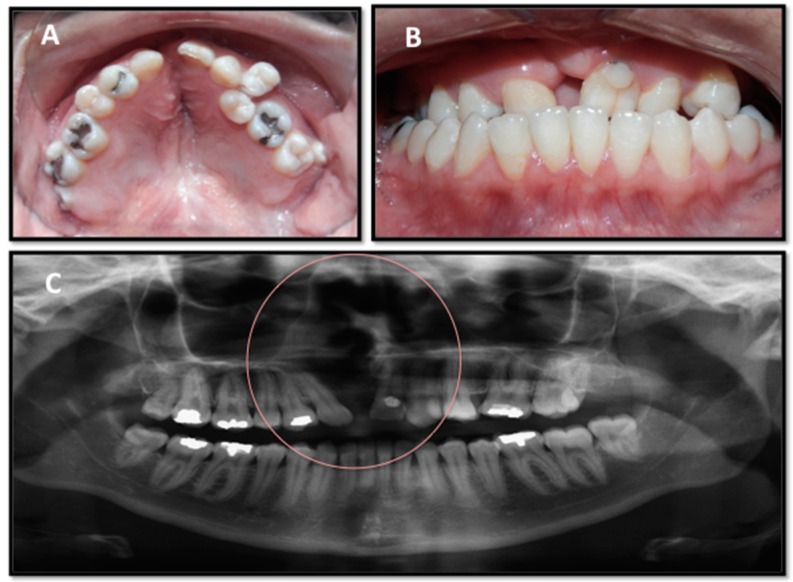
Image of a patient with unilateral cleft palate showing the different tissues involved (bone, dental organs, respiratory system and soft tissue) that need to be attended during the treatment and some malformation around the orofacial area responsible for causing respiratory and speech/language problems. Deformation of the arch and dental crowding (**A**), crossbite dental malposition (**B**), and the deviated nasal septum (**C**) as revealed by panoramic radiographs showing the maxillary defect (circle) (unpublished data).

The management of patients with CL/P pathology is complex and requires a multidisciplinary approach that includes plastic surgeons, maxillofacial surgeons (cleft surgeons), otolaryngologists, speech/language pathologists, audiologists, dentists, orthodontists, psychologists, geneticists, and social workers. Different tissues including bone, dental organs, and soft tissue from the respiratory system are largely affected during the CL/P reconstruction (Figure 3), therefore it is necessary to standardize the perioperative management of these patients [17].

Regarding the reconstruction of alveolar cleft defects, the most accepted approach consists of the secondary alveolar cleft osteoplasty in the mixed dentition phase [10]. The goal of this surgery is to achieve a normal facial appearance as well as the ability to feed, speak, and hear without affecting the ultimate facial appearance of the child. To achieve this goal, the most common palatoplasty techniques currently accepted are the von Langenbeck technique, the Bardach 2-flap palatoplasty, the Veau–Wardill–Kilner closure, the 2-stage palatoplasty, and the Furlow palatoplasty [1]. Ultimately, there is also variability on the optimal timing to perform palate repair. As transverse facial growth is not completed until five years of age, some surgeons have considered retarding cleft palate repair, even to as late as age 8 or 10, to reduce the risk of midface hypoplasia, while others may consider an earlier repair before the age of two, in order to improve speech development and achieve better integration in society with less psychosocial impact for the children and families. Taking the middle position, some surgeons have managed cleft palate repair in two stages, with soft palate repair at three to six months and hard palate repair at 15 to 18 months, while others have advocated a single-stage repair with both the soft and hard palates being repaired simultaneously. Unfortunately, none of these surgeries are definitive and may present long-term complications including palatal fistula, velopharyngeal insufficiency, and midface hypoplasia resulting in facial growth disturbance in multiple dimensions and cross bite abnormalities such as transverse maxillary hypoplasia that need to be managed by orthodontic maxillary expansion with fixed appliances and supported by bone grafting in order to consolidate the dental arch and teeth alignment [1,18].

Nowadays, the use of autogenous bone is the most widely used type of grafting in bone regeneration defects [2,19]. However, the availability of autogenous bone is limited and is not free of tremendous drawbacks, especially in pediatric patients where the availability for harvesting bone may be limited and thus may not be the ideal graft for alveolar bone reconstruction. In itself, this process is usually invasive and has the potential for significant morbidities to occur at the donor site, such as infection, paresthesia, postoperative pain and scarring problems [19,20]. As an alternative, tissue engineering strategies offer the possibility of using artificial custom made supports for tissues and cells with the aim for them to be applied in the affected area to promote the regeneration of missing or damaged tissues.

The current bioartificial tissues designed for cleft palate reconstruction have been mostly based on inserted granules isolated with a single tissue layer [10,21]. However, the alveolar cleft defect typically consists of a two-wall bony defect in which mucoperiosteal flaps are sutured in two layers to create a new nasal floor and a continuous oral mucosa. As a consequence, the free motion of the inserted granules negatively affects the dimensional stability and biomechanical properties of the reconstructed sites, difficulty with the correct closure of these mucoperiosteal flaps, and isolation from microorganisms that can infect the graft [22]. In order to overcome these limitations, the most sophisticated approaches to CL/P repair consider the fabrication of biomodels with a 3D shape and microstructure similar to patients’ bone defects to test the biomechanical properties of bone substitutes and evaluate the clinical effects with respect to osteogenesis and healing, first in vitro and second in experimental animals. Several animal models have been utilized for the testing of alveolar cleft grafting materials including mice, rabbits, cats, dogs, goats, sheep, and monkeys, with rats being the most referred model among them due to their ease of handling and cost effectiveness. However, these defects made on rats are significantly smaller in volume than human alveolar defects, thus it is difficult to extrapolate the results [8,23]. In order to overcome these limitations, according to Pourebrahim et al., artificial biomodels created in experimental animals had to fulfill the following criteria: there had to be a bilateral maxillary alveolar cleft with a 15 mm bony width in each research animal, with demonstrable oronasal communication, covered by healthy epithelialized mucosa; and there must be functional teeth on each side [10].

Some authors have also evaluated in vivo genetically induced CL/P models in rats. It was described that due to a sevoflurane-induced gene deletion, an incomplete development of the palate and alveolus was achieved. However, in many cases, the gene defect led to other pathologies and perinatal lethality, therefore, this methodology has been considered as not suitable to evaluate new bone grafts [17,24].

### Stem Cells Alternative and Growth Factor Assisted Regeneration

**Figure 4:**
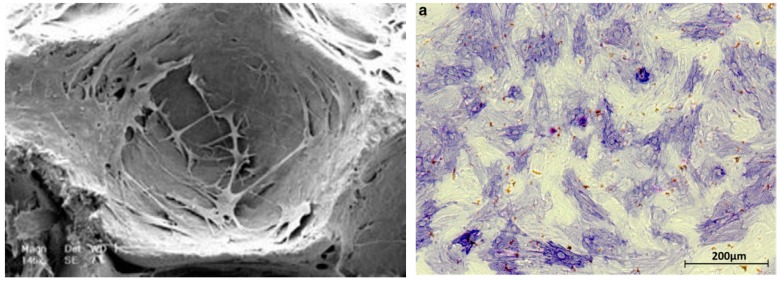
(**Left**) Scanning electron microscope views of the HA/TCP scaffolds Ceraform^®^ seeded with Adipocyte stem cells (ADSCs) used for human maxillofacial reconstruction showing the ability of ADSC to adhere on the surface of and colonize the inner pores of the scaffolds. (**Right**) Alkaline phosphatase analysis of osteogenically differentiated BMSC cells after three days of cultivation on bovine hydroxyl apatite/collagen scaffolds. Reproduced from Pourebrahim et al. (2013) [10] and Korn et al. (2017) [24] with permission from Elsevier and Springer^®^, respectively.

Adult stem cells are considered fundamental for cell therapy because of their unique ability to self-renew and differentiate into various phenotypes, in addition to being obtained from different tissues and have been used for craniofacial defect regeneration in tissue engineering. Adipocyte stem cells (ADSCs) are particularly desirable candidates for musculoskeletal tissue engineering applications such as cleft lip and palate [10]. In this sense, Pourebrahin et al. proposed the use of adipose tissue in maxillary alveolar cleft defects, due to their potential for differentiation, the easy accessibility to this source of cells, and their capability to rapidly expand in vitro. The authors studied the potential of ADSCs seeded in biphasic bone substitutes of hydroxyapatite/calcium triphosphate (HA/TCP) to repair maxillofacial bone defects (Figure 4) in a dog model, concluding that they were an acceptable alternative for the reconstruction of human maxillofacial bone defects in the case of limited autograft availability or morbidity in the donor site [10].

Complementary to ADSCs, another source of adult mesenchymal stem cells can be isolated from bone marrow (BMSC) and dental pulp (HDPSC). There have been multiple examples of maxillofacial bone regeneration using these sources of cells. Korn et al. demonstrated that BMSCs could be used to promote bone formation in a maxillary defect through their osteogenic differentiation mediated by BMP-4 (Figure 4) [24], and more recently, Al-Ahmady et al. introduced a novel strategy for alveolar cleft reconstruction by combining BMSCs seeded on a collagen sponge with platelet-rich fibrin (PRF) and nano-hydroxyapatite [20].

PRF is a platelet concentrate, as a source of growth factors basically used to enhance soft and hard tissue healing and has been used in plastic and maxillofacial surgery, in addition to many tissue engineering models [25,26,27,28]. Its advantages include ease of preparation, application, and absence of chemical alteration. Additionally, previous studies have shown that PRF growth factors were released in a time-dependent manner, resulting in prolonged biological effects [29]. In addition, the fibrin network of the PRF allows cell migration of endothelial cells essential for angiogenesis, neurogenesis, vascularization and subsistence of the graft at the site of regeneration.

This is why PRFs have been present as a strong alternative and presumably cost-effective biomaterial for maxillofacial tissue repair and CL/P regeneration [27].

## 3. Biomaterials for Soft and Hard Cleft Tissue Repair

Biomaterials play a key role in the tissue engineering strategy for the restoration of missing tissue and its functionality. In particular, the advances in bone regeneration using biomimetic 3D scaffolds made of bioceramics, polymers, and composites, using different manufacturing methods (i.e., 3D printing, cryopolymerization, synthesis, etc.), have permitted the exploration of new options for the repair of tissues in CL/P treatment.

### 3.1. Bioceramics

**Figure 5:**
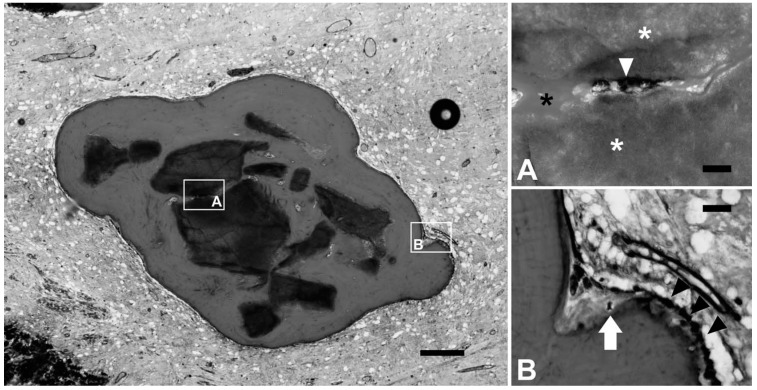
(**Left**) Induced bone formation by beta-TCP in the maxillary cleft of goats (**A**). Material (stars) is reabsorbed by a multinucleated osteoclast-like cell (arrowhead) (**B**). Elsewhere, cuboidal osteoblasts (black arrow heads) lay down new bone (pink) adjacent to an osteocyte (white arrow) in its lacuna. Reproduced from Janssen et al. (2017) [22] with permission from SAGE Publications ^®^. Scale bars: 250 μm (left), 25 μm (right **A**, **B**).

Bioceramics such as hydroxyapatite (HA), α-tricalciumphosphates (αTCP) and β-tricalciumphosphates (βTCP), demineralized bone matrices, calcium carbonates, calcium sulfates, bioactive glasses, and composite materials in combination with bioactive inorganic materials (bioglasses, etc.) constitute an important group of biomaterials used to manufacture adequate scaffolds in relation to novel treatments for CL/P due to their desired biological properties in terms of osteoconduction, biocompatibility, chemical similarity with natural bone and facilitate proliferation and osteoblast differentiation [30,31]. Janssen et al. described osteoinductive microstructured βTCP granules, embedded in a glycerol matrix, as an alternative to autologous bone grafts for alveolar cleft repair because of their ability to induce bone formation when implanted at heterotopic sites in a bilateral alveolar goat cleft model. These authors hypothesized that the quality of residual bone and the volume of the putty would work at least equal to the autograft and, even, the surgical management would be superior to the use of the regular β-TCP granules (Figure 5) [22]. Contrary to these findings, Korn et al. showed that when using hydroxyapatite/collagen composite scaffolds, the ossification of the defect was not enhanced, probably due to the micromovements of the remaining non-resorbable HA particles after their degradation of the collagen that hampered, as in the case of autografts, the ossification of the defects. Nevertheless, most of the investigations using scaffolds based on bioceramics are supported by cell therapy and growth factors and although the osteoinduction mechanism has not yet been completely revealed, the relationship between the physical and chemical features of the osteoinductive bioceramic and the osteogenic differentiation of HMSCs and their suitability for craniofacial defect repair including alveolar cleft palate regeneration has been demonstrated [8,17,19,21,25].

### 3.2. Polymeric Biomaterials

**Figure 6:**
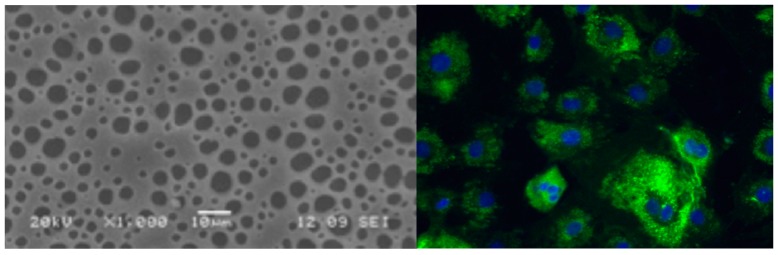
Human dental pulp stem cells seeded in multiwall carbon nanotubes with PCL at day 21 with potential application in CL/P regeneration. Osteopontin labeled antibody was used to evaluate the expression of bone phenotype markers, nuclei were counter stained with DAPI (unpublished data). Scale bars: 10 μm (left), 100 μm (right).

Recent advances in macromolecular sciences and tissue engineering methods have made it possible to efficiently generate several human artificial tissues including the oral mucosa and maxillofacial bone such as cleft palate [32]. Several synthetic polymer scaffold materials have been used for these purposes including poly (ε-caprolactone) (PCL), poly(lactic acid) (PLA), poly(glycerol sebacate) (PGS), poly (lactide-co-glycolide) (PLGA), or polyhydroxyalkanoates (PHA), among others [33]. These polymers can be synthesized in large quantities under controlled conditions, thus ensuring uniform and reproducible properties while reducing the risks of infections and immunogenicity [34]. For example, Flores-Cedillo et al. prepared membrane composites made of multiwall carbon nanotubes (MWCNTs) with PCL, demonstrating their ability to allow adhesion and proliferation of human dental pulp stem cells (HDPSCs) (Figure 6), and promoting their osteogenic differentiation toward bone like phenotypes permitting bone regeneration, and thus suitable for CL/P regeneration.

A new generation of advanced 3D polymeric scaffolds has resulted in very promising results. Hoshi et al. developed an implant-type tissue-engineered cartilage using a PLA based scaffold and evaluated it clinically by inserting it into subcutaneous areas of nasal dorsum in three patients to correct cleft lip–nose deformity. Subsequently, one year after implantation, the maintenance of the morphology in the dorsum and apex of the nose of the patients was confirmed [35]. Similar results were also reported by Puwanun et al. but using biodegradable electrospun PCL scaffolds with the ability to support bone-forming cells and within cleft palate bone defects [36]. Moreover, these scaffolds can be developed by incorporating hybrid natural derived biomaterials such as collagen or chitosan, that in combination with PCL and PLGA copolymer nanofibers serve to offer scaffolding options with superior osteogenic potential by combining the biomimetic and stimulating effects of natural polymers and the structural and mechanical stability capabilities of synthetic polymers [37,38,39,40,41]. On this note, an alternative strategy proposed by Zaky et al. aimed to enhance biocompatibility, biodegradability, and material elasticity by creating a biomimetic cellular niche based on poly glycerol sebacate (PGS) in which bone marrow stromal cells were mechanically stimulated to produce their own extracellular matrix leading to a biochemically mimicking environment of bone, while enabling the transmission of mechanical forces with the objective of treating craniofacial malformations including CL/P [42].

## 4. New Manufacturing Techniques for Cleft Palate Reconstruction

**Figure 7:**
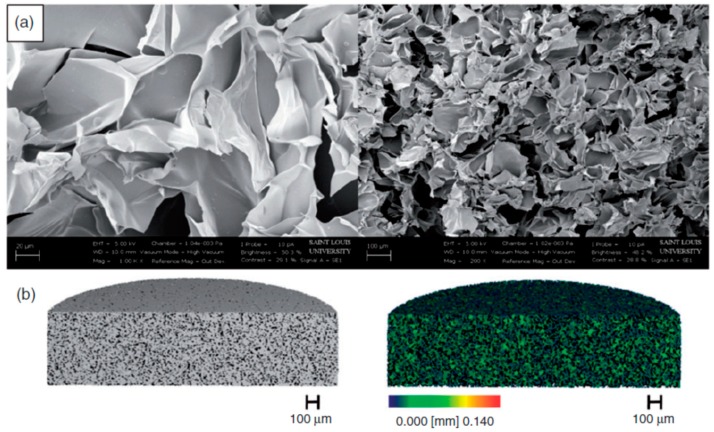
Analysis of a patient custom made patient cryogel. (**a**) SEM images taken at 1000 and 200X (left to right). (**b**) mCT 3D reconstruction images representing both the scaffold (grey) and the inner pores with the color bar denoting the size of the pores within the cryogel (left to right). Reproduced from Hixon et al. (2017) [45] with permission from SAGE^®^.

Some of the most challenging difficulties for craniofacial defect regeneration are derived from the variety of tissue-specific requirements and the complexity of anatomical structures in that region [43,44]. Thus, hierarchical micro-structured and custom-made scaffolds are often required for regenerative therapies. Fortunately, the current advances in the fabrication of in situ click-chemistry based injectable formulations, controlled cryopolymerization methods, electrospinning, and 3D direct printing of complex structures with composite biomaterials are able to provide scaffolds with adequate nano-, micro- and macro-structure and composition for CL/P repair. On this note, Hixon et al. described cryogel scaffolds as tissue-engineered constructs formed at sub-zero temperatures, with excellent potential for the treatment of patient-specific bone defects (Figure 7). In addition, these authors used patient-specific 3D-printed molds derived from computed tomography for scaffold fabrication during the thawing of the cryogels, resulting in a macroporous, sponge-like, and mechanically durable product for the creation of site-specific implants in the treatment of patients with CL/P [45].

## 5. Folic Acid Derivatives as Osteoinductive Molecules for Cleft Palate Regeneration

Maternal folic acid during the periconceptional period is considered to be one of the main causes of clefting disorders. A recent review published by Fernandez Villa et al. [46] highlighted the potential of folic acid as a key bioactive compound to enhance the effectiveness of biomaterial performance and biological functions for the regeneration of tissues and organs. In addition, new derivatives of folic acid bearing bioactive cations such as Sr or Zn have been proven to be promising compounds with the ability to accelerate bone formation in craniofacial defects [47] and reduce inflammation [48].

**Figure 8:**
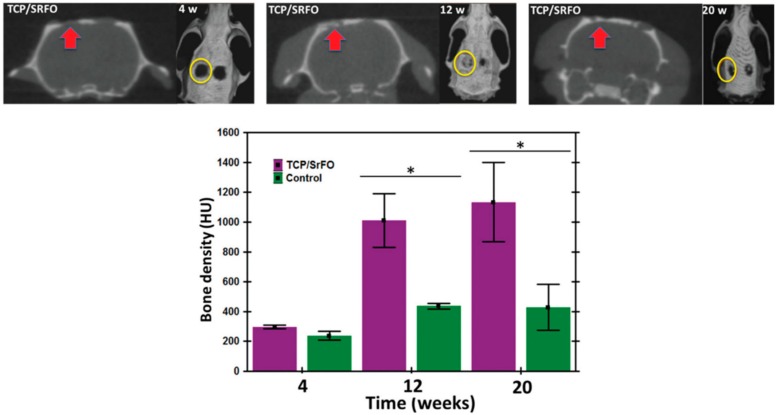
Micro-computed tomography images of cranial defects treated with TCP/SrFO scaffolds at 4, 12, and 20 weeks, and defect closure on the side of the implants form the coronal plane (arrows) and 3D images (circles) and bone density of the radiographic density (HU) in cranial defects. (* = Significant differences *p* < 0.001). Reproduced from [39] with permission from the Royal society for Chemistry.

The therapy based on Sr seems promising due to its proven action in improving preosteoblast replication, osteoblast differentiation, synthesis of collagen type I, and mineralization of the bone matrix. Nonetheless, any formulation should provide an effective and consistent way to deliver Sr^2+^ ions with low or the absence of secondary pharmacological effects. In this regard, Rojo et al. developed a carrier for Sr based on folic acid with a remarkable capacity of enhancing bone tissue formation and synergic benefits on cell replication and differentiation processes. In agreement with these authors, Martín-del-Campo et al. demonstrated that the incorporation of strontium folate within 3D porous bio-hybrid scaffolds provided an excellent system for the regeneration of bone tissue into the craniofacial area (Figure 8) [39]. The use of these strontium folate derivatives, in combination with HDPSC and biomimetic scaffolds, is a promising alternative that can be used at accessible cost for bone regeneration, in particular during CL/P treatment.

## 6. Conclusions and Future Perspectives

The success of synthetic bone grafts is based on their capacity to promote osteoconductivity and osteoinductivity during the formation of new bone growth. In addition, the use of low molecular weight compounds such as those derived from folic acid and bioactive cations constitutes a promising alternative to the use of protein-based growth factors and morphogens, for the preparation of resorbable scaffolds in the maxillary defect model to allow osteoconduction and osteoinduction in the defects. In this regard, the use of bioceramics such as calcium phosphate in combination with biomimetic polymer scaffolds, folic acid derivatives, morphogens, and stem cells are currently considered as the most promising alternative for CL/P regeneration. In addition, emerging bioprinting technologies in combination with advanced manufacturing techniques such electrospinning or cryogelation processes have permitted the development of new tissue substitutes with a precise control of sizes and shapes to recreate the complex physiological, biomechanical, and hierarchical microstructure of biological tissues that are necessary for the regeneration of malformations such as CL/P.
